# The Involvement of MiR-1-Clathrin Pathway in the Regulation of Phagocytosis

**DOI:** 10.1371/journal.pone.0098747

**Published:** 2014-06-16

**Authors:** Cuilian Liu, Jiajia Wang, Xiaobo Zhang

**Affiliations:** Key Laboratory of Animal Virology of Ministry of Agriculture and College of Life Sciences, Zhejiang University, Hangzhou, The People's Republic of China; Thomas Jefferson University, United States of America

## Abstract

Phagocytosis, one of the most powerful immune responses, is a complicated process regulated by many factors. However the regulation of phagocytosis mediated by microRNAs has not been extensively investigated. To address this issue, the regulation of phagocytosis by miR-1 was characterized in this study. The results showed that miR-1 played an important role in the phagocytosis regulation in shrimp *in vivo*. The sequence analysis indicated that miR-1 was highly conserved from invertebrates to mammals, suggesting that miR-1 might share the similar or same functions in phagocytosis of shrimp hemocytes and mammalian macrophages. The data presented that miR-1 was significantly downregulated in cancerous macrophage RAW264.7 cells compared with those in the isolated murine macrophage and in the immortalized macrophage ANA-1. The findings showed that miR-1 had a great effect on the regulation of phagocytosis in cancerous macrophage by the inhibition of clathrin heavy chain 1 (*CLTC1*) gene. Therefore our study presented a novel miR-1-mediated regulation of phagocytosis both in invertebrate and in vertebrate.

## Introduction

Phagocytosis, a phylogenetically conserved process in organisms, is critical for innate immune response. The professional phagocytes such as macrophage and polymorphonuclear granulocytes can transport large particles into intracellular compartments or phagosomes by internalization. The engulfment of particles by phagocytes is triggered by their interactions with specific receptors on the phagocyte surfaces, which results in the remodeling of actin and the extension of membrane protrusions to form a closed phagosome. In phagocytes, the invaded microorganisms, cell debris and diverse particulates are degraded [1], [2]. During the phagocytosis process, a lot of regulators, such as transmembrane proteins, actin binding proteins, membrane traffic regulators and ion channel proteins, are involved in the regulation of phagocytosis [1], [3]–[5]. As reported, the myosin light chain kinase, which is phosphorylated and activated by ERK, plays important roles in the regulation of phagocytosis by the activation of cytoskeletal components [6]. The engagement of SIRPβ can promote phagocytosis in macrophages by inducing the tyrosine phosphorylation of DAP12, Syk and SLP-76 [7]. However, the interaction between SHPS-1 and CD47 on macrophages initiates the tyrosine phosphorylation of SHPS-1 and thereby prevents the FcγR-mediated disruption of the SHPS-1-SHP-1 complex, resulting in the inhibition of phagocytosis [8]. In inflammatory responses, the macrophage phagocytosis may be rapidly modulated in response to extracellular environmental signals, such as cAMP [9]. The upregulation of macrophage capacity for phagocytosis represents an important aspect of therapeutic strategies for the modulation of inflammatory diseases [9]. It is revealed that macrophages play critical roles in the response of the central and peripheral nervous systems to injury and disease by the component receptor-3 (CR3/MAC-1)-mediated myelin phagocytosis [10].

Except for proteins, miRNAs are found to play critical modulating roles during the phagocytosis process in recent years. As reported in our previous study, 12 miRNAs are found to be related to the regulation of phagocytosis in shrimp [11]. It is revealed that microRNA-125a-5p takes great effects on the regulation of inflammatory response in monocytes/macrophages [12]. In macrophages, miR-155 is upregulated in response to lipopolysaccharide (LPS), suggesting its involvement in phagocytosis [13]. Three miRNAs miR-34a, miR-155 and miR-326 can downregulate the expression of CD47 in the lesion environment [14]. As a result, macrophages are released from inhibitory control and phagocytosis is promoted. As reported, miR-146 can control Toll-like receptor (TLR) and cytokine signaling by targeting the IRAK-1 and TRAF6, two essential components of the TLR signaling pathways, acting as a negative regulator of immune response [15]. Increasing evidence supports the involvement of microRNAs in the regulation of inflammatory and immune processes including phagocytosis. To date, however, the roles of miRNAs and their corresponding pathways in phagocytosis have not been intensively explored.

Based on our previous study [11], the role of miR-1 in the regulation of phagocytosis was characterized in this investigation. The results showed that miR-1 could regulate phagocytosis by targeting the clathrin mRNA. The findings expanded our knowledge about the modulating role of miRNA in innate immunity.

## Results

### 1. The regulation of phagocytosis mediated by miR-1 in shrimp

**Figure 1:**
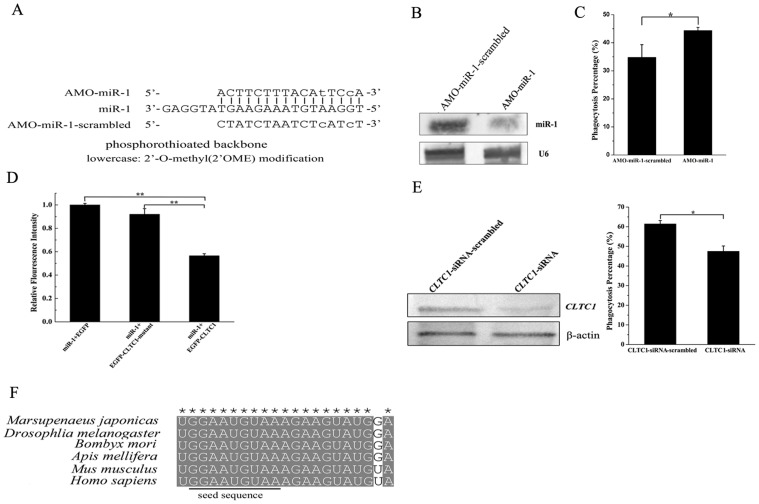
The regulation of phagocytosis mediated by miR-1 in shrimp. (A) Nucleotide sequences and modifications of the anti-miRNA-1 oligonucleotide (AMO-miR-1) and AMO-miR-1-scrambled. (B) Silencing of miR-1 expression in shrimp hemocytes *in vivo*. The miR-1-specific AMO (AMO-miR-1) and the negative control AMO-miR-1-scrambled were injected into shrimp, respectively. The shrimp hemocytes were subjected to Northern blot using miR-1 or U6 probe. (C) The effect of miR-1 expression silencing on phagocytic activity of shrimp hemocytes. The phagocytic activity of FITC-labeled WSSV was evaluated with flow cytometry. The plotted data points referred to the means ± standard deviations of triplicate assays and the asterisk represented statistically significant differences (*, *p*<0.05). (D) The interaction between miR-1 and *CLTC1* gene. The miR-1 precursor and the plasmid EGFP-*CLTC1* or EGFP-*CLTC1*-mutant or EGFP were cotransfected into insect High Five cells. Then the fluorescence intensity of cells was monitored with a fluorescence microscope. The columns represented the means ± standard deviations of triplicate assays. The significant differences between treatments were indicated with asterisks (**, *p*<0.01). (E) The effect of *CLTC1* on phagocytosis of shrimp hemocytes. The shrimp were injected with *CLTC1*-siRNA or *CLTC1*-siRNA- scrambled as a control. Then the shrimp were subjected to Northern blots (left) and phagocytosis assays (right). In Northern blots, the shrimp β-actin was used as a control. The significant differences between treatments were indicated with asterisk (*, *p*<0.05). (F) Sequence alignment of miR-1 from six typical species. * indicated the identical nucleotides.

Our previous study revealed that miR-1 was upregulated when the phagocytic activity of shrimp hemocytes was inhibited, indicating the involvement of miR-1 in the regulation of phagocytosis [11]. To further characterize the role of miR-1 in phagocytosis, the expression of miR-1 was silenced using miR-1-specific anti-miRNA oligonucleotide (AMO) in shrimp *in vivo* (Fig. 1A). Northern blots showed that the expression of miR-1 was knocked down in shrimp hemocytes compared with the control (AMO-miR-1-scrambled) (Fig. 1B). In the case of miR-1 expression silencing, the phagocytic activity of shrimp hemocytes was evaluated with FITC-labeled white spot syndrome virus (WSSV) *in vivo*. The loss-of-function data indicated that the phagocytosis percentage of miR-1-silenced hemocytes was significantly increased compared with that of the control (AMO-miR-1-scrambled) (Fig. 1C), showing that the downregulation of miR-1 led to the increase of phagocytic activity. The findings presented that miR-1 played important roles in the regulation of phagocytosis in shrimp *in vivo*.

The target predictions indicated that the clathrin heavy chain 1 (*CLTC1*) gene might be a target gene of miR-1 in shrimp. To evaluate the interaction between miR-1 and *CLTC1* gene, the miR-1 precursor and the plasmid EGFP-*CLTC1* or EGFP-*CLTC1*-mutant were cotransfected into insect High Five cells. The results demonstrated that the fluorescence intensity in cells treated with miR-1 and EGFP-*CLTC1* was significantly decreased compared with the controls (Fig. 1D), showing that miR-1 could target the *CLTC1* gene.

To characterize the effect of *CLTC1* on phagocytosis of shrimp hemocytes, the expression of *CLTC1* was silenced by *CLTC1*-siRNA, followed by phagocytosis assay. Northern blots indicated that the expression of *CLTC1* gene was knocked down (Fig. 1E). The silencing of *CLTC1* gene expression resulted in a significant decrease of phagocytosis percentage of shrimp hemocytes by comparison with the control (Fig. 1E), indicating that *CLTC1* was involved in phagocytosis in shrimp.

The sequences of miR-1 from different species of animals were aligned. The results showed that the miR-1 sequence was highly conserved in 6 typical species from invertebrates to the highest mammalian *Homo sapiens* (Fig. 1F). The sequence conservation of miR-1 suggested that miR-1 might be involved in the regulation of phagocytosis in mammals as revealed in shrimp.

### 2. Effects of miR-1 on phagocytosis of mammalian macrophages

**Figure 2:**
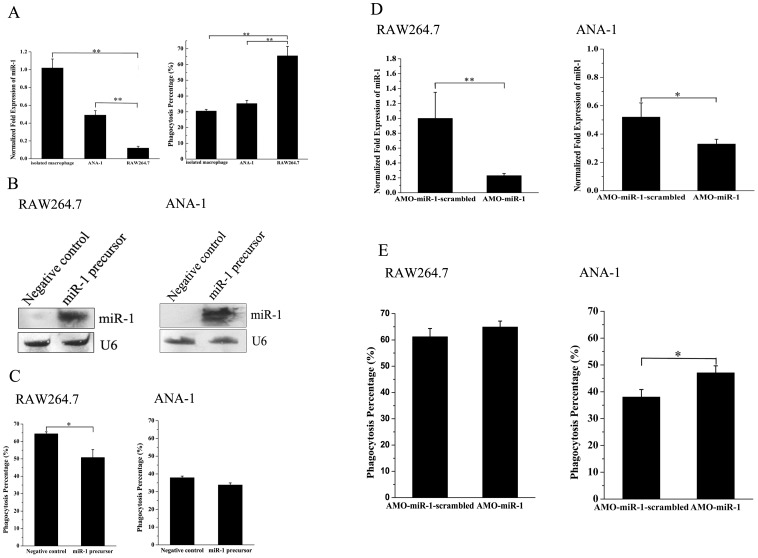
The role of miR-1 in the regulation of phagocytosis in mammalian macrophages. (A) Expression levels of miR-1 and phagocytic activities in the isolated murine macrophage, the immortalized macrophage ANA-1 and the cancerous macrophage RAW264.7. The expression of miR-1 was quantified with real-time PCR (left). The phagocytic activity was evaluated using FITC-labeled *E. coli* (right). The data referred to the means ± standard deviations of triplicate assays. (B) Overexpression of miR-1 in RAW264.7 and ANA-1 cells. Cells were transfected with miR-1 precursor or control miRNA. At 24 h after transfection, the total RNAs were isolated from transfected cells and subjected to Northern blot. The expression level of miR-1 was normalized with U6. (C) Evaluation of phagocytic activities in RAW264.7 and ANA-1 cells against FITC-labeled *E. coli* by flow cytometry. Cells transfected with miR-1 precursor or control miRNA were challenged with FITC-labeled *E. coli*, followed by phagocytosis assays. (D) Knockdown of miR-1 in RAW264.7 and ANA-1 cells. The cells were transfected with AMO-miR-1 or AMO-miR-1-scrambled. Twenty four hours later, the total RNAs were isolated from transfected cells. The expression of miR-1 was determined by quantitative real-time PCR. (E) Phagocytosis percentages of RAW264.7 and ANA-1 cells treated with AMO-miR-1 or AMO-miR-1 -scrambled. At 24 h after transfection of AMOs, the phagocytosis was evaluated using FITC-labeled *E. coli*. In all panels, plotted data points referred to the means ± standard deviations of triplicate assays and asterisks represented statistically significant differences (**, *p*<0.01; *, *p*<0.05).

To characterize the roles of miR-1 in phagocytosis of mammalian macrophages, the expressions of miR-1 in the cancerous macrophage RAW264.7, the immortalized macrophage ANA-1 and the isolated murine macrophage were evaluated. The results showed that the expression level of miR-1 was significantly upregulated in cancerous macrophages compared with those in the isolated murine macrophage and the immortalized macrophage ANA-1 (Fig. 2A), suggesting that miR-1 might play important roles in the regulation of phagocytosis in cancerous macrophage. As assayed, the miR-1 expression level in the isolated macrophages was around twice higher than that in the immortalized macrophages (Fig. 2A). This differential expression of miR-1 might result from the immortalization process. It was revealed that the phagocytic activity of RAW264.7 cells was higher than those of ANA-1 cells and the isolated macrophages (Fig. 2A).

Due to the downregulation of miR-1 in the cancerous macrophage RAW264.7, the gain-of-function experiments of miR-1 were conducted to evaluate the effects of miR-1 overexpression on phagocytosis of macrophages. The precursor of miR-1 was transfected into RAW264.7 and ANA-1 cells, respectively. The Northern blot results showed that miR-1 was overexpressed in RAW264.7 and ANA-1 cells (Fig. 2B). It was revealed that the overexpression of miR-1 led to a significant decrease of phagocytic percentage of RAW264.7 cells against FITC-labeled *E. coli* (Fig. 2C). After the transfection of miR-1 precursor, however, the phagocytic percentage was unchanged in ANA-1 cells (Fig. 2C). These findings showed that the overexpression of miR-1 resulted in the inhibition of phagocytic activity in cancerous macrophage, but it had no effect on phagocytosis of normal macrophage.

For a comprehensive evaluation of the effects of miR-1 on phagocytosis in cancerous macrophage, the expression of miR-1 was knocked down using the anti-miRNA oligonucleotide (AMO) AMO-miR-1. The results indicated that the miR-1 expression was specifically silenced in RAW264.7 and ANA-1 cells by AMO-miR-1 (Fig. 2D). The phagocytosis assays revealed that the silencing of miR-1 in ANA-1 cells resulted in a significant increase of phagocytic percentage compared with the control AMO-miR-1-scrambled (Fig. 2E). However, the knockdown of miR-1 did not take any effect on the phagocytic activity of RAW264.7 cells.

### 3. The mechanism of phagocytosis regulation mediated by miR-1

**Figure 3:**
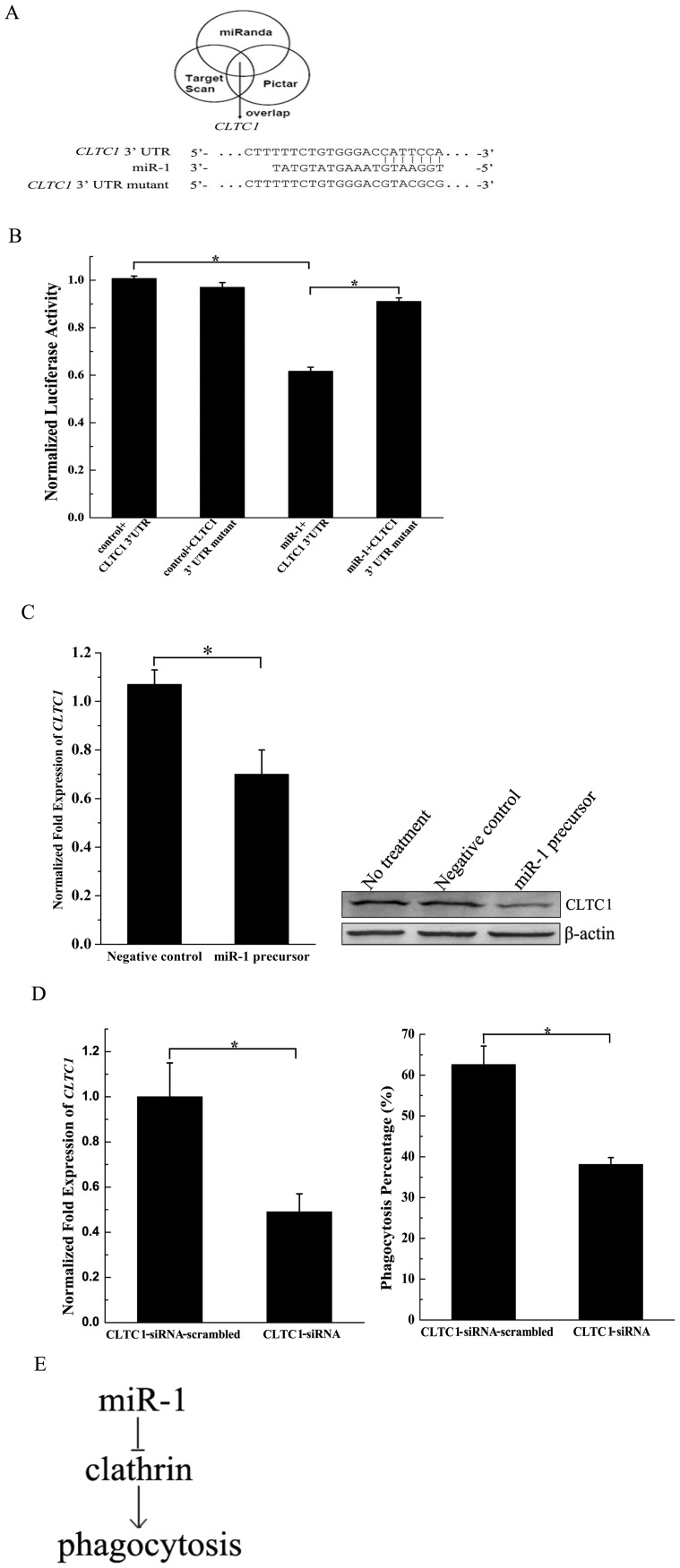
The mechanism of phagocytosis regulation mediated by miR-1. (A) miR-1 targets analysis. Clathrin heavy chain 1 gene (*CLTC1*) was a predicted target gene of miR-1. (B) The interaction between miR-1 and *CLTC1* gene. RAW264.7 cells were simultaneously transfected with miR-1 or control (plasmid only) and the *CLTC1* gene 3′ UTR, followed by a dual luciferase reporter assay. The *CLTC1* gene 3′ UTR mutant was included in the assays. (C) Downregulation of endogenous *CLTC1* gene by miR-1 in RAW264.7 cells. The miR-1 precursor and the negative control were transfected into RAW264.7 cells, respectively. Subsequently the *CLTC1* mRNA was detected using real-time quantitative PCR (left) and the *CLTC1* protein was examined with Western blot (right). In real-time PCR, the expression level of *CLTC1* gene was normalized to that of glyceraldehyde-3-phosphate dehydrogenase gene. In Western blot, the antibodies used were indicated on the right. (D) The role of *CLTC1* in phagocytosis of macrophages. RAW264.7. Cells were transfected with *CLTC1*-siRNA to silence the expression of *CLTC1*. *CLTC1*-siRNA-scrambled was used as a control. At 48 h after transfection, the expression of *CLTC1* was detected with quantitative real-time PCR (left). At the same time, the phagocytosis percentages of RAW264.7 cells were evaluated using flow cytometry (right). (E) Model for miR-1-mediated pathway in phagocytosis. In all panels, the data referred to the means ± standard deviation of triplicate assays. Statistically significant differences between treatments were indicated with asterisk (*, *p*<0.05).

To reveal the mechanism of the miR-1-mediated phagocytosis regulation, the targets of miR-1 were predicted using computational strategies. Based on target predictions, the clathrin heavy chain 1 (*CLTC1*) gene might be a target gene of miR-1 (Fig. 3A). The results showed that the seed sequence of miR-1 was complementary to the 3′ untranslated region (UTR) of *CLTC1* gene.

The dual-luciferase reporter assays were conducted in RAW264.7 cells to characterize the interaction between miR-1 and the 3′ UTR of *CLTC1* gene. The results showed that the luciferase activity of the treatment miR-1+*CLTC1* 3′ UTR was significantly decreased compared with those of the controls (control+*CLTC1* 3′ UTR and miR-1+*CLTC1* 3′ UTR mutant) (Fig. 3B), showing that miR-1 inhibited the expression of *CLTC1* gene. However, miR-1 took no effect on the expression of *CLTC1* 3′ UTR mutant (Fig. 3B). There was no difference between controls (Fig. 3B). The miR-1 precursor and its negative control was transfected into RAW264.7 cells, followed by the detection of *CLTC1* gene transcript. The data demonstrated that the expression of *CLTC1* gene was significantly decreased by miR-1, but not by the negative control (Fig. 3C). The results indicated that miR-1 played an important role in the expression regulation of *CLTC1* gene in macrophages.

To examine the role of *CLTC1* in phagocytosis, the *CLTC1* gene expression was silenced by *CLTC1*-siRNA, followed by phagocytosis assay. The results showed that the expression of *CLTC1* was knocked down by *CLTC1*-siRNA, while the control (*CLTC1*-siRNA-scambled) took no effect on the *CLTC1* expression (Fig. 3D). The silencing of *CLTC1* expression led to a significant decrease of phagocytosis percentage of RAW264.7 cells compared with the control (Fig. 3D). The data presented that *CLTC1* played a positive role in phagocytosis.

Considering all the above data, miR-1 was involved in phagocytosis by inhibiting the expression of *CLTC1* gene (Fig. 3E).

## Discussion

Phagocytosis, one of the most powerful ways to eliminate invading pathogens in innate immunity, is conserved in vertebrates and invertebrates [16]. During the phagocytosis process, many proteins are found to be involved in its regulation [1], [3]–[5]. In recent years, the roles of microRNAs in the regulation of phagocytosis have attracted more and more investigations. However, the mechanism of miRNA- mediated regulation of phagocytosis is not intensively studied. On the basis of our previous investigation [11], in this study, the results revealed that miR-1, a sequence-conserved miRNA in animals, took great effects on the negative regulation of phagocytosis of shrimp hemocytes and murine macrophage RAW264.7 cells. Our findings indicated that the sequence-conserved miRNA in vertebrates and invertebrates might share similar or same functions in animal immunity. Invertebrates including shrimp lack a true adaptive immune response system and have developed an effective non-specific innate immune response for detecting and eliminating noxious microorganisms. As reported, phagocytosis plays an essential role in shrimp immunity [17], [18]. In this context, shrimp could be served as a good candidate to reveal the regulation of phagocytosis mediated by miRNAs.

It is reported that microRNAs can regulate diverse biological processes by targeting the mRNAs of target genes [19]. In this study, the data presented that miR-1 was involved in the regulation of phagocytosis through the interaction between miR-1 and the 3′ UTR of clathrin heavy chain 1 (*CLTC1*) gene. Clathrin consists of three heavy and three light chains. Clathrin-coated vesicles are mainly involved in mediating internalization of many cell surface proteins from the plasma membrane and returning some of them through recycling endosomes back to the plasma membrane [20]. In phagocytosis, the occupied receptors on cell surface of phagocyte activate a signaling cascade that leads to actin polymerization, plasma membrane remodeling, and extension of pseudopods around the particles [2]. The receptor/membrane recycling takes place through a clathrin-mediated endocytic pathway. During the phagocytosis process, clathrin can be recruited to the phagocytic cup in modest amount [21], [22]. The recruitment of clathrin to the cup may have a covert purpose to participate in the fission of vesicles from the phagosome during phagosome maturation [23], [24]. The previous study shows that clathrin plays an important role in phagocytosis. In this study, the results indicated that the downregulation of *CLTC1* by sequence-specific siRNA resulted in a significant decrease of phagocytic activity in macrophages, showing the involvement of clathrin in phagocytosis. In this context, the miR-1-clathrin interaction revealed in this investigation represented a novel mechanism of the regulation of phagocytosis.

## Materials and Methods

### 1. Ethics Statement and Data Availability

Animal experiments were carried out in strict accordance with the recommendations in the Guide for the Care and Use of Laboratory Animals of the National Institutes of Health. The protocol was approved by the Committee on the Ethics of Animal Experiments of Zhejiang University. All surgery was performed under sodium pentobarbital anesthesia, and all efforts were made to minimize suffering. All data are available upon request.

### 2. Shrimp culture

*Marsupenaeus japonicus* shrimp (5–6 g/shrimp and 6 cm/shrimp) were raised in seawater with salinity of 26 g/L and pump ventilation at 20°C. For each treatment, shrimp were kept in a group of 20 individuals.

### 3. Cell culture

The murine macrophage cell line RAW264.7, derived from a BALB/c mouse tumor induced by Abelson leukemia virus [25], and the cell line ANA-1, obtained by immortalization of C57BL/6 mouse bone marrow cells [26], were purchased from the Type Culture Collection of the Chinese Academy of Sciences, Shanghai, China. The cells were cultured in RPMI 1640 medium (Gibico, USA) supplemented with 10% fetal bovine serum (FBS) in a humidified atmosphere of 5% CO_2_ and 95% air at 37°C.

High Five (Hi 5) insect cells were cultured following standard procedures (Invitrogen, USA). Cells were grown at 27°C in GIBCO insect culture medium (Invitrogen) supplemented with 5% heat-inactivated FBS (Thermo, USA).

### 4. Loss-of-function assay of miR-1 in shrimp *in vivo*

To silence the expression of miR-1, the anti-miRNA-1 oligonucleotide (AMO-miR-1) was injected into shrimp at 0.144 mM/shrimp for twice at an interval of 12 h. As a control, the sequence of AMO-miR-1 was scrambled, yielding AMO-miR-1-scrambled. The AMO-miR-100-scrambled (0.144 mM/shrimp) was included in the injections. At 24 h after the last injection, the hemolymph specimens collected from three shrimp, selected at random, were mixed and subjected to Northern bolt and phagocytosis assays. All the assays described above were biologically repeated for three times.

### 5. Northern blot analysis

Total RNAs were extracted from shrimp hemocytes or human cell lines using mirVana miRNA isolation kit (Ambion, USA) and then treated with RNase-free DNase I (Ambion). The extracted RNAs were separated by electrophoresis on a 15% polyacrylamide gel containing 7M urea for 2 h. Subsequently the RNAs were transferred to a nylon membrane (Amersham Biosciences, UK), followed by UV cross-linking. The blots fixed on the nylon membrane were probed with digoxigenin (DIG)-labeled miR-1 (5′- ATACATACTTCTTTACATTCCA-3′) or DIG-labeled U6 (5′-GGGCCATGCTAATC TTCTCTGTATCGTT-3′) probe. The detection for DIG was performed using anti- DIG-alkaline phosphatase (Roche, Switzerland) for 2 h at room temperature. After washing three times for 5 min each, the membrane was incubated with the substrate BCIP/NBT solution (Roche).

For the detection of *CLTC1* mRNA, the total RNAs were extracted from shrimp hemocytes. The RNAs were separated by agarose gel electrophoresis and then detected with the DIG-labeled *CLTC1* (5′-AAGGTCTGGATTCTGTAGCACATTG GTGAT-3′) or DIG-labeled β-actin (5′-CACGCCATCGCCAGAGTCCAGCACG- 3′) as described above.

### 6. Phagocytosis assay of shrimp hemocytes

Shrimp hemocytes were exsanguinated with phosphate buffered saline (PBS) containing 20 mM EDTA (ethylene diamine tetraacetic acid). The hemocytes were centrifuged at 500×g for 5 min and washed twice with PBS containing 20 mM EDTA. Then the hemocytes were incubated with inactivated FITC (fluorescein isothiocyanate) -labeled WSSV (white spot syndrome virus) virions for 30 min at 28°C [17]. After washes twice with PBS, the hemocytes were fixed with 1% formaldehyde in PBS on ice. The phagocytosis percentage of FITC-positive hemocytes was evaluated using flow cytometry.

### 7. Fluorescence assays

To evaluate the interaction between miR-7 and shrimp *CLTC1* gene, fluorescence assays were performed in insect High Five cells. The shrimp *CLTC1* and the enhanced green fluorescent protein (EGFP) genes were cloned into a pIZ/EGFP V5-His vector (Invitrogen, USA) using gene-specific primers (5′- AATTC CGCCTGGCTCAGAACT-3′ and 5′-GTAGTACAGTTCAATGTTGGCAAC-3′) to make EGFP-*CLTC1* construct. As a control, the seed sequence of shrimp *CLTC1* was randomly mutated and cloned into pIZ/EGFP V5-His vector using primers 5′- AATTCCGCCTGGCTCAGAACT-3′ and 5′- TCTGGTCCAGGGTTAACGCCTT A -3′ to construct EGFP-*CLTC1*-mutant. When the High Five cells were at about 70% confluence, they were cotransfected with the miR-1 precursor (30 nM) and a plasmid containing the EGFP gene or EGFP-*CLTC1* or EGFP-*CLTC1*-mutant (2 µg/mL) using Cellfectin transfection reagent according to the manufacturer's protocol (Invitrogen, USA). At 48 h after transfection, the fluorescence of the cells was monitored with a Flex Station II microplate reader (Molecular Devices, USA) at 490 and 510 nm for excitation and emission. The fluorescence values were corrected by subtracting the autofluorescence of cells not expressing EGFP. All the experiments were repeated biologically three times.

### 8. Preparation of siRNAs and RNAi assays in shrimp

Based on the sequence of *CLTC1* gene, *CLTC1*-siRNA (5′-GCAGAUCUAGA GGAGUUUA-3′) specifically targeting the *CLTC1* gene was designed by BLOCK-iT RNAi Designer (https://rnaidesigner.lifetechnologies.com/) and synthesized using the in vitro transcription T7 kit for siRNA synthesis (TaKaRa, Japan) according to the manufacturer's instructions. As a control, the sequence of *CLTC1*-siRNA was scrambled (*CLTC1*-siRNA-scrambled, 5′-GCAUACAGUGAUG UAUGAG-3′). The synthesized siRNAs were dissolved in siRNA solution (50 mM Tris-HCl, 100 mM NaCl, pH 7.5) and quantified by spectrophotometry. The siRNAs were injected into the lateral area of the fourth abdominal segment at 30 µg/shrimp using a syringe with a 29-gauge needle. Three shrimp from each treatment, selected at random, were collected at 36 h after the siRNA injection and subjected to Northern blotting and phagocytosis assay. All assays were repeated three times.

### 9. Sequence alignment analysis

The sequences of miR-1 from six typical species including *Marsupenaeus japonicas*, *Drosophlia melanogaster*, *Bmobyx mori*, *Apis mellifera*, *Mus musculus* and *Homo sapiens* were obtained from miRbase (http://www.mirbase.org/). The conserved nucleotides of miR-1 were analyzed with sequence alignment.

### 10. Isolation of murine spleen macrophages

Mice were sacrificed by cervical dislocation and their spleens were isolated for the isolation of macrophages [27]. The spleens were cut into small pieces and grinded in RPMI-1640 medium (Gibico, USA) on ice. After passing through 200 mesh cell sieve, the spleen cell suspension was collected. Subsequently mononuclear cells were isolated from the spleen cell suspension by density gradient centrifugation with 70% Percoll (Sangon Biotech Co., Ltd, Shanghai, China). The isolated mononuclear cells were plated in RPMI-1640 medium (Gibico, USA) at 3×10^5^ cells per well and incubated at 37°C in a humidified atmosphere of 5% CO_2_ in air. One hour later, the nonadherent cells were removed by washing three times with PBS. More than 90% of adherent cells were macrophages.

### 11. Quantification of miRNA by real-time PCR

Total RNAs were extracted from macrophages with mirVana miRNA isolation kit (Ambion, USA). After treatment with DNase I, the cDNA was reversely transcribed from the total RNA using miRNA-specific primer with TaqMan microRNA reverse transcription kit (Applied Biosystems, USA). Then real-time PCR was conducted at 95°C for 10 min, followed by 50 cycles at 95°C for 15 s and 60°C for 1 min. The real-time PCR reaction mixture (10 µl) contained 0.5 µl of RT product, 5 µl of TaqMan 2×Universal PCR Master Mix (Applied Biosystems), 1 µl TaqMan miRNA Assay (Applied Biosystems). U6 (Applied Biosystems) was used as an internal standard for normalization.

### 12. Silencing and overexpression of miR-1 in mammal macrophage cell lines

RAW264.7 and ANA-1 cells were respectively transfected with 30 nM of miR-1 precursor (Applied Biosystems, USA) to overexpress miR-1 in mammal macrophage cells. The negative control miRNA (30 nM) was included in the transfections. The miR-1 precursor and the negative control were purchased from Applied Biosystem (USA). To silence the expression of miR-1 in RAW264.7 and ANA-1 cells, the cells were transfected with 50 nM of anti-miRNA oligonucleotide (AMO) AMO-miR-1 or the control AMO-miR-1-scrambled. The sequence of AMO-miR-1 (5′-ACTTCTTTA CA**T**TC**C**A-3′) was modified with 2′-O- methyl (bold) and phosphorothioate (the remaining nucleotides). The sequence of AMO-miR-1 was randomly scrambled, generating AMO-miR-1-scrambled (5′-TCTACTCTAAT**C**TA**T**C-3′) with the same modifications as above. The AMO-miR-1 and AMO-miR-1-scrambled were synthesized by Sangon Biotech (Shanghai) Co., Ltd (Shanghai, China). The AMOs and miRNA precursor were transfected into cells with Lipofectamine RNAiMAX (Invitrogen, USA) following the manufacturer's protocol. At 24 h after transfections, the total RNAs were extracted from the transfected cells and subjected to Northern blot and quantitative real-time PCR analysis.

### 13. Phagocytosis assays of mammal macrophages

At 24 h after transfection with AMOs, miR-1 precursor or control miRNA, the cells at 3∼5×10^5^ cells/ml were washed twice with PBS. Then the cells were added with FITC-labeled *E. coli* at a ratio of 10∶1 and incubated for 30 min at 37°C and 5% CO_2_. The cells were washed to remove the unbound FITC-labeled *E. coli*, followed by quenching with trypan blue for 5 min. Subsequently the cells were washed three times and resuspended with 1% paraformaldehyde. The phagocytosis percentage of cells was evaluated using flow cytometry.

### 14. Prediction of target genes of miR-1

The potential target genes of miR-1 were predicted using miRanda, TargetScan and Pictar [28]–[30]. The overlapped target genes were further investigated.

### 15. Dual-luciferase reporter assays in RAW264.7 cells

Dual-luciferase reporter assays were performed in RAW264.7 cells to evaluate the interaction between miR-1 and its target gene 3′ untranslated region (3′ UTR). The 3′ UTR of clathrin heavy chain 1 gene was amplified by RT-PCR using primers 5′-C*GA GCTC*GATGGAAGCTGATCCTGT-3′ (SacI, italic) and 5′-GG*CTCGAG*CTCTTCT GTACACTCAGT-3′ (XhoI, italic). Then it was cloned into pmirGLO Dual- Luciferase miRNA Target Expression Vector (Promega, USA) and confirmed by DNA sequencing. As a control, the seed sequence of clathrin heavy chain 1 gene 3′ UTR was randomly mutated and cloned into pmirGLO Dual-Luciferase miRNA Target Expression Vector to construct its mutant (5′-CTTTTTCTGTGGGACGTACG CC-3′). The mutant sequence was confirmed by DNA sequencing. The miR-1 (5′-UG GAAUGUAAAGAAGUAUGUAU-3′) in miExpress vector was obtained from Applied Biosystem (USA).

The RAW264.7 cells were co-transfected with miR-1 and clathrin heavy chain 1 gene 3′ UTR or the mutant of clathrin heavy chain 1 gene 3′ UTR using Attractene Transfection Reagent (Qiagen, USA). At 24 h after transfection, the cells were collected and washed twice with PBS. Then the passive lysis buffer (Promega, USA) was added to promote rapid lysis of cells. After incubation for 15 min at room temperature, the lysate was used for luciferase activity detection. The luciferase activity detection was performed according to the protocol of Dual-Luciferase Assay Reagent (Promega, USA). All the experiments were repeated for three times.

### 16. Quantitative real-time PCR analysis of *CLTC1* gene expression in RAW264.7 cells

RAW264.7 cells were transfected with 30 nM of miR-1 precursor (Ambion, USA) or the negative control of miR-1. At 24 h after transfection, the cells were collected and total RNAs were extracted with mirVana miRNA isolation kit (Ambion, USA), followed by the treatment with RNase-free DNase I. The first-strand cDNA of *CLTC1* was synthesized by reverse transcription with PrimeScript 1st strand cDNA synthesis kit (Takara, Japan). Then real-time PCR was conducted to evaluate the expression of *CLTC1* using primers 5′- GTGGTAATCATTG ATATGAATG-3′ and 5′-GTGACATCATCAGTCATG-3′ and TaqMan probe 5′-FAM-CGAAGACCA ATCTCAGCAGACAGT-TAMRA-3′. The expression levels were normalized to glyceraldehyde-3-phosphate dehydrogenase (GAPDH). The expression of GAPDH gene was monitored with gene-specific primers (5′-CAATGTGTCCGTCGTGGATC T-3′ and 5′-GTCCTCAGTGTAGCCCAAGATG-3′) and TaqMan probe (5′-FAM- CGTGCCGCCTGGAGAAACCTGCC-TAMRA-3′).

### 17. Western blotting

Cells were collected by centrifugation at 300×g for 10 min and completely lysed by radio immunoprecipitation assay (RIPA) buffer (Beyotime, China) containing 2 mM phenylmethanesulfonyl fluoride (PMSF). The samples were denatured, separated on SDS-PAGE, and then electrotransfered to a polyvinylidene fluoride (PVDF) membrane (Millipore, USA) by electrophoresis for 45 min at 200 V. After protein transferring, the blot was incubated in blocking solution [4% bovine serum albumin (BSA) in TBST (Tris-buffered saline and Tween-20)] overnight, followed by incubation with primary antibody for 2 h at 4°C. Subsequently the membrane was incubated with AP-conjugated secondary antibody (Roche) for 2 h at room temperature. The membrane, after washes with TBST, was then incubated with BCIP/NBT substrate (Sangon, China) until the blot was visualized. The primary antibodies against CLTC1 and β-actin were purchased from Cell Signal Technology (USA).

### 18. Silencing of the expression of *CLTC1* in RAW264.7 cells

RAW264.7 cells were transfected with 100 nM of *CLTC1*-siRNA (5′-GCTGTC AGATCATCAATTA-3′) or *CLTC1*-siRNA-scrambled (5′-ATCGTACATGTACTAG CTA-3′) as a control. The siRNAs were designed and synthesized by GenePharma (Shanghai, China). The siRNAs were transfected into cells with Lipofectamine RNAiMAX according to the manufacturer's protocol (Invitrogen, USA). At 48 h after transfections, the transfected cells were used for quantitative real-time PCR analysis and phagocytosis assay.

### 19. Statistical analysis

The numerical data were analyzed by one-way analysis of variance to calculate the mean and standard deviation of triplicate assays. The T test was conducted to analyze the differences between treatments.
